# Candida vaginitis among symptomatic pregnant women attending antenatal clinics in Mwanza, Tanzania

**DOI:** 10.1186/s13104-019-4793-z

**Published:** 2019-11-27

**Authors:** Martha F. Mushi, Amani Mmole, Stephen E. Mshana

**Affiliations:** Department of Microbiology and Immunology, Catholic University of Health and Allied Sciences, P. O. Box 1464, Mwanza, Tanzania

**Keywords:** Candida vaginitis, *Candida albicans*, Douching, Antibiotic use, Low social economic status

## Abstract

**Objective:**

This study was done to determine the patterns of *Candida* spp. causing vaginitis and associated factors among pregnant women attending antennal clinic in Mwanza, Tanzania.

**Results:**

A total of 197 (65.6%) out of 300 non-repetitive swabs had positive growth of *Candida* spp. *Candida albicans* 125 (63.4%) was the most predominant isolated specie followed by *C. tropicalis 35* (17.8%) and *C. glabrata* 33 (16.8%). Laboratory confirmed candida vaginitis was independently predicted by douching practices (OR 3.2, 95% CI 1.3–7.5 P = 0.007), history of antibiotics use (OR 1.8, 95% CI 1.02–3.0, P = 0.04) and low social economic status (OR 2.04, 95% CI 1.1–3.7 P = 0.02). About two-third of pregnant women with clinical features of vaginitis attending antenatal clinic in Mwanza, Tanzania were confirmed to have Candida vaginitis mainly caused by *Candida albicans*.

## Introduction

Vulvo vaginal candidiasis or Candida vaginitis is the fungal infection of the female lower genital tract (vagina and vulva) caused by *Candida* spp. [1]. Candida vaginitis is the second most complain among women attending obstetrics and gynecological clinics worldwide [2, 3]. It is estimated that about 75% of women are affected with Candida vaginitis at least once during their life time with 15% of these cases present with a “cyclic recurrent type” which is defined as four or more episodes of Candida vaginitis in a year [4, 5].

Studies have shown that *Candida albicans* accounts for 80–95% of all episodes of candida vaginitis worldwide [4, 6]. However, there is an increase of cases due to non-*Candida albicans* species led by *Candida glabrata* [7]. Other non-*Candida albicans* species reported to be associated with Candida vaginitis include: *Candida tropicalis*, *Candida parapsilosis*, *Candida lusitaniae*, *Candida famata*, *Candida kefyr*, *Candida sake*, *Candida inconspicua*, *Candida valida*, *Candida colliculosa*, *Candida utilis*, *Candida catenulata*, *Candida lipolytica*, *Candida membranaefaciens*, *Candida intermedia* and *Candida globosa* [7–9].

In East Africa, a study done in Aghakhan hospital-Kenya reported *C. albicans* as the prominent species with prevalence of 69.3% followed by *C. glabrata* 12.9% [10]. In Tanzania, non-*Candida albicans* species were reported to contribute about 37% of Candida vaginitis cases [11]. However, data on azole susceptibility patterns and factors associated with Candida vaginitis are still limited. Here, we report the prevalence and factors associated with laboratory confirmed Candida vaginitis among pregnant women with symptoms of vaginitis attending antenatal clinics in Mwanza, Tanzania. Furthermore, data on the azole susceptibility patterns of these *Candida* spp. are reported.

## Main text

This was a cross section study conducted from February to July 2016. The study was conducted at antenatal clinics of Nyamagana district hospital and Makongoro reproductive and child health clinic in Mwanza, Northwestern Tanzania. The selected clinics are representative of antenatal clinics that are highly populated in Mwanza serving more than 100 pregnant women per day.

Fungal isolation and fungal speciation by chromogenic agar were carried in the CUHAS microbiology laboratory and specie confirmation and antifungal susceptibility testing was done at Institute of Medical Microbiology, Gottingen, Germany.

All pregnant mothers attending antenatal clinics suspected of having Candida vaginitis and consented were recruited serially until the sample size was reached. For this study clinical Candida vaginitis was defined as having two or more of the following symptoms vaginal pruritus (itching), a thick odorless cottage cheese-like discharge and soreness [1]. The minimum sample size was obtained by the use of Kish Leslie formula [4].

### Culture for isolation and identifications of *Candida* spp.

High vaginal swabs were cultured on Sabouraud’s dextrose agar (SDA) supplemented with 50 µg/ml gentamicin and 50 µg/ml chloramphenicol (HiMedia-Mumbai, India) as previously described [12]. All yeasts isolated were identified to species level using chromogenic agar. Furthermore, 98 randomly picked isolates were confirmed by the use of matrix-assisted laser desorption ionization-time of flight (MALDI-TOF) mass spectrometry (Bruker Daltonics, Bremen, Germany) on extracted cells harvested from SDA as previous described [12, 13].

### In vitro susceptibility assays

Antifungal susceptibility testing was done by establishing minimum inhibitory concentration (MIC) of fluconazole, voriconazole, posaconazole (Discovery Fine Chemicals, Bournemouth, United Kingdom), micafungin (Roth, Germany), caspofungin (Merck, US), and 5-fluorocytosine (Sigma Aldrich, US) following the guidelines laid down by the EUCAST [13].

### Data analysis and management

All data collected were entered into Microsoft excel sheet for cleaning and coding then transferred to STATA version 13 for analysis according to the objectives of the study. Data were summarized into percentage for categorical variables while continuous variables (age and gestation age) were summarized as median with interquartile range (IQR). Logistic regression analysis was done to determine predictors of candida vaginitis. Statistical significant was considered when P value was less than 0.05 with 95% confidence interval.

### Ethical considerations

The protocol to conduct this study was approved by the joint Catholic University of Health and Allied Sciences/Bugando Medical Centre research ethics and review committee (CREC) with certificate number CREC/045/2014. Permission to conduct the study was sought from all hospital administrations. All patients were requested to sign the written informed consent before recruitment and patients’ data were treated as confidential.

## Results

A total of 300 pregnant women with mean age of 27 ± 6.2 years were recruited. The majority of women were married 275 (91.7%), resided in urban areas 262 (87.3%)  and had primary school education 201 (67%). About half of studied participants 168 (56%) booked their first antenatal clinic on third trimester while 106 (35.3%) and 26 (8.7%) booked on second and first trimester, respectively. Their median gestation age at recruitment was 28 with interquartile range of 20–32 weeks. Most of the pregnant women had low social economic status 221 (74%) as defined by having a fridge and television.

**Fig. 1:**
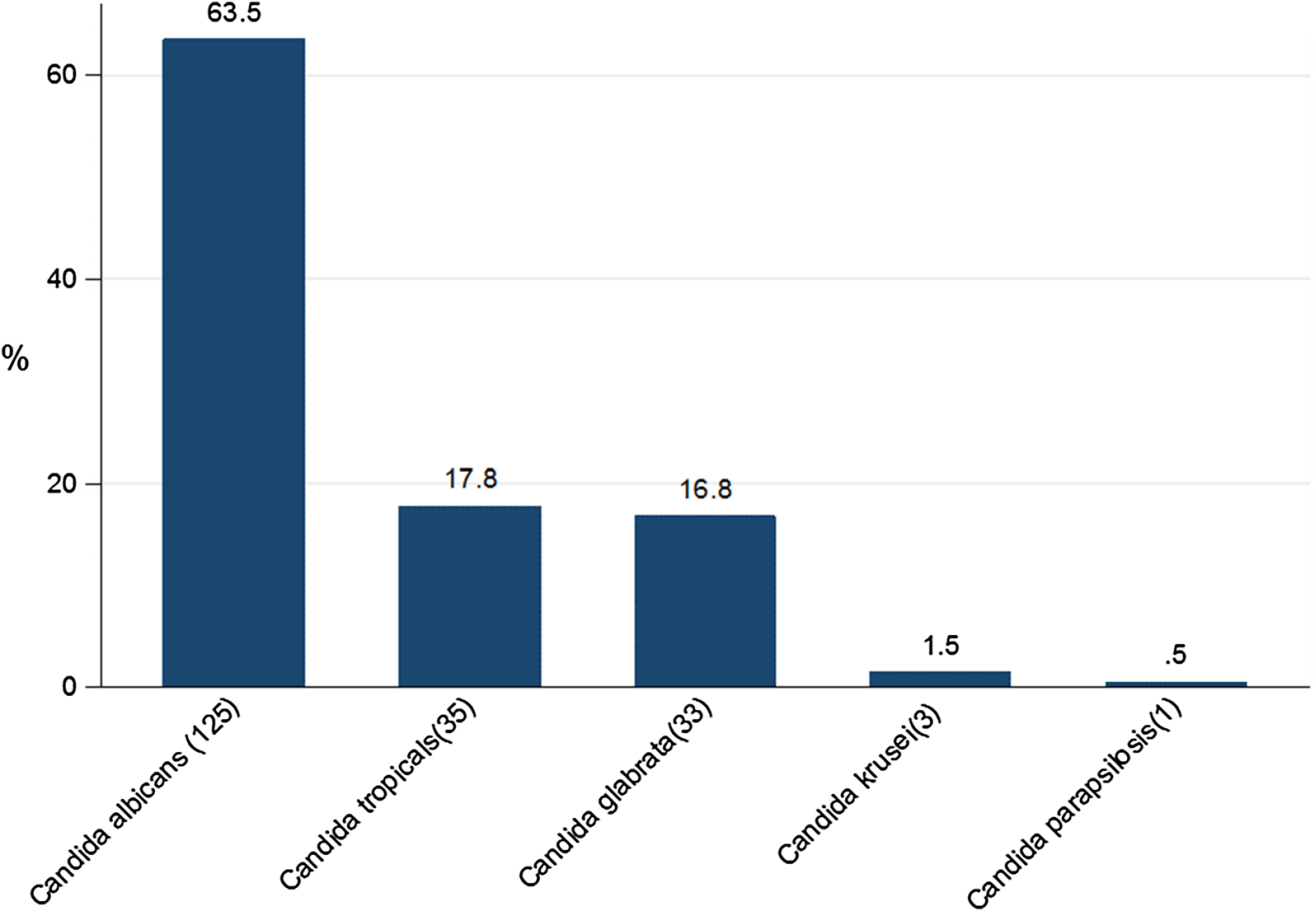
Distributions of *Candida* spp. detected to cause Candida vaginitis

Laboratory confirmed Candida vaginitis was detected in 65.7% (197/300) of symptomatic pregnant women. *Candida albicans* was the most predominant detected *Candida* spp. 125 (63.4%). A total of 72 (24%) patients were diagnosed to have Candida vaginitis caused by non-*Candida albicans* spp. The predominant non *Candida albicans* spp. detected was *Candida tropicalis* 35 (17.8%), Fig. 1. All isolated *Candida albicans* were highly susceptible to azole antifungal agents. However, *Candida krusei* were highly resistant to fluconazole and susceptible to other azole agents, Additional file 1.

### Factors associated with Candida vaginitis among pregnant women

On univariable analysis, increase in gestation age OR 1.03, 95% CI 1.01–1.06, P = 0.04, not having a secondary school education OR 2, 95% CI 1.3–3.3, P = 0.008, practicing douching OR 2.8, 95% CI 1.28–6.25, P = 0.01, history of antibiotic use in the past 2 weeks OR 1.8, 95% CI 1.15–3.03, P = 0.011 and low social economic status OR 2.03, 95% CI 1.21–3.42, P = 0.008 were found to be associated with laboratory confirmed Candida vaginitis, Table 1.

**Table 1:** Factors associated with vaginal Candida vaginitis among 300 studied pregnant women

Variable	Culture positive (%)	Culture negative (%)	Univariate		Multivariate	
			OR (95% CI)	P value	OR (95% CI)	P value
Age	28 [22–32]	26 [22–30]	1.03 (1.0–1.07)	0.119	1.01 (0.9–1.1)	0.528
Gestation age	28 [20–32]	24 [20–32]	1.03 (1.01–1.06)	0.041	1.03 (0.9–1.1)	0.068
Residence						
Urban (262)	167 (63.7)	95 (36.3)	1			
Rural (38)	30 (78.9)	8 (21.1)	0.46 (0.21–1.06)	0.070	–	–
Marital status						
Single (25)	14 (56.0)	11 (44.0)	1			
Married (275)	183 (66.6)	92 (33.4)	1.56 (0.68–3.58)	0.291	–	–
Education						
Primary (201)	141 (70.1)	60 (29.9)	1			
Secondary (89)	48 (53.9)	41 (46.1)	2 (1.3–3.3)	0.008	1.25 (0.8–2.5)	0.411
College (10)	8 (80.0)	2 (20)	1.7 (0.4–8.3)	0.5.9	3.5 (0.6–18.9)	0.150
Occupation						
Employed (184)	114 (67.0)	70 (38.0)	1			
Unemployed (116)	83 (71.6)	33 (28.5)	1.54 (0.93–2.54)	0.089	–	–
Yoghurt drinking						
Yes (119)	72 (60.5)	47 (39.5)	1			
No (181)	125 (69.1)	56 (30.9)	1.46 (0.89–2.36)	0.128	–	–
Drinking alcohol						
No (255)	166 (65.1)	89 (34.9)	1			
Yes (45)	31 (68.9)	14 (31.1)	1.18 (0.6–2.3)	0.622	–	–
Gravidity						
Prime gravid (73)	43 (58.9)	30 (41.1)	1			
Gravid two (78)	51 (65.4)	27 (34.6)	1.3 (0.6–2.5)	0.412	–	–
Multi gravid (149)	103 (69.1)	46 (38.9)	1.56 (0.9–2.8)	0.133	–	–
Douching						
No (28)	12 (42.9)	16 (57.1)	1			
Yes (272)	185 (68.0)	87 (32.0)	2.8 (1.28–6.25)	0.01	3.2 (1.4–7.5)	0.007
STI results*						
Negative (233)	159 (68.2)	74 (31.8)	1			
Positive (10)	3 (30)	7 (70)	5.01 (1.3–19.7)	0.022	4.6 (1.1–19.9)	0.039
Antibiotic use						
No (147)	86 (58.5)	61 (41.5)	1			
Yes (153)	111 (72.6)	42 (27.4)	1.8 (1.15–3.032)	0.011	1.8 (1.02–3.0)	0.020
HIV						
Negative (290)	189 (65.2)	101 (34.8)	1			
Positive (10)	8 (80.00)	2 (20.0)	2.14 (0.45–10.3)	0.342	–	–
SES						
High (82)	44 (53.7)	38 (46.3)	1			
Low (218)	153 (70.2)	65 (29.8)	2.03 (1.21–3.42)	0.008	2.04 (1.1–3.7)	0.02

The box bracket [] is inter quartile range for gestation age while the curved brackets () is percentage

*Only 243 women tested for VDRL

*SES* Social economic status

On multivariable logistic regression analysis, having douching practices OR 3.2, 95% CI 1.4–7.5, P = 0.007, history of antibiotic use in the past 2 weeks OR 1.8, 95% CI 1.02–3.0, P = 0.02 and low social economic status OR 2.04, 95% CI 1.1–3.7, P = 0.02 were independent predictors of laboratory confirmed Candida vaginitis, Table 1.

## Discussion

Candida vaginitis or thrush is the vaginal infection caused by yeast cells and commonly being reported among pregnant women of 20–40 years of age [2]. This has also been observed in the current study were by the median age of the pregnant women with clinical Candida vaginitis was 28 with interquartile range of 22–32 years. Physiological and tissue changes, due to reproductive hormones, which happen in young women especially during pregnancy, increase their susceptibility to Candida infection, in addition to adverse factors such as risky sexual behaviors. Previous study conducted in North America suggested that 70–75% of women can get at least one episode of Candida vaginitis in life time [6].

The current study established the prevalence of laboratory Candida vaginitis to be 65.7% among symptomatic pregnant women. This finding is similar to previous report from Nigeria which reported the prevalence of 62.2% [4] and slightly higher than 55.4% that was reported in Cameroon [14]. The different in study population could explain the differences. The study in Cameroon involved health non-pregnant women while the current study and the one from Nigeria involved pregnant women. The changes in sex hormones during pregnancy has been highly associated with the increases chances of Candida vaginitis [3].

As previous reported from different studies [2, 3, 11], the current study found *C. albicans* to be the most predominant specie causing Candida vaginitis. The virulence nature of *Candida albicans* in comparison to other *Candida* spp. could explain the findings [13]. Furthermore, *Candida albicans* has been reported to be the most common *Candida* spp. colonizing the vaginal mucosa giving it high chance of causing infections in case of the presence of favorable conditions [10].

The use of antibiotic is known to suppress the bacterial normal flora and allow the overgrowth of yeast cells hence causing Candida vaginitis [15]. This was proven in the current study whereby the use of antibiotic was found to be an independent predictor of laboratory confirmed Candida vaginitis. Additionally in the current study douching practices which is also known to impair the growth of the vaginal microbiota [16–18] was found to independently predict the laboratory confirmed Candida vaginitis.

In the current study having low social economic status was also found to predict laboratory confirmed Candida vaginitis. Low social economic status is associated with poor hygiene [19]. Inability of the women to access basic needs include clean water and health care affect their hygienic practices. The poor hygienic practices can easily lead to vaginal candidiasis as previous observed in Cameroon [3].

About two-third of pregnant women with clinical features of vaginitis attending antenatal clinic in Mwanza, Tanzania were laboratory confirmed to have Candida vaginitis mainly caused by Candida albicans. Pregnant women with low social economic status (SES) with history of antibiotic use and who are practicing douching are more likely to suffer Candida vaginitis. A large cohort study among the high risk groups is recommended to determine the effect of the vaginal candidiasis to the pregnancy.

## Limitation

The prevalence of laboratory Candida vaginitis in the current might been under estimated because the features used are not specific for Candida vaginitis.

## Supplementary information

**Additional file 1: Table S1.** Antifungal susceptibility test data.

## Data Availability

The datasets used and/or analyzed during the current study available from the corresponding author on reasonable request.

## Abbreviations

BMC: Bugando Medical Centre
CUHAS: Catholic University of Health and Allied Sciences
EUCAST: European Committee of Antimicrobial Susceptibility Testing
OR: odd ratio
MALDI-TOF: Matrix-assisted Laser Desorption Ionization-Time of Flight
SDA: Sabouraud’s dextrose agar
